# Laser triangulation measurements of scoliotic spine curvatures

**DOI:** 10.1186/s13013-015-0050-y

**Published:** 2015-09-01

**Authors:** Dušan Čelan, Breda Jesenšek Papež, Primož Poredoš, Janez Možina

**Affiliations:** University of Maribor, Faculty of Medicine, Taborska ulica 8, 2000 Maribor, Slovenia; University Medical Centre Maribor, Ljubljanska ulica 5, 2000 Maribor, Slovenia; University of Ljubljana, Faculty of Mechanical Engineering, Aškerčeva 6, 1000 Ljubljana, Slovenia

**Keywords:** Scoliosis, 3D measurement, Spinous process, Spinal curvature, Cranio-caudal view

## Abstract

**Background:**

The main purpose of this research was to develop a new method for differentiating between scoliotic and healthy subjects by analysing the curvatures of their spines in the cranio-caudal view.

**Methods:**

The study included 247 subjects with physiological curvatures of the spine and 28 subjects with clinically confirmed scoliosis. The curvature of the spine was determined by a computer analysis of the surface of the back, measured with a non-invasive, 3D, laser-triangulation system. The determined spinal curve was represented in the transversal plane, which is perpendicular to the line segment that was defined by the initial point and the end point of the spinal curve. This was achieved using a rotation matrix. The distances between the extreme points in the antero-posterior (AP) and left-right (LR) views were calculated in relation to the length of the spine as well as the quotient of these two values LR/AP. All the measured parameters were compared between the scoliotic and control groups using the Student’s *t*-Test in case of normal data and Kruskal-Wallis test in case of non-normal data. Besides, a comprehensive diagram representing the distances between the extreme points in the AP and LR views was introduced, which clearly demonstrated the direction and the size of the thoracic and lumbar spinal curvatures for each individual subject.

**Results:**

While the distances between the extreme points of the spine in the AP view were found to differ only slightly between the groups (*p* = 0.1), the distances between the LR extreme points were found to be significantly greater in the scoliosis group, compared to the control group (*p* < 0.001). The quotient LR/AP was statistically significantly different in both groups (*p* < 0.001).

**Conclusions:**

The main innovation of the presented method is the ability to differentiate a scoliotic subject from a healthy subject by assessing the curvature of the spine in the cranio-caudal view. Therefore, the proposed method could be useful for human posture diagnostics as well as to provide a long-term monitoring of scoliotic spine curvatures in preventive and curative clinical practice at all levels of health care.

## Background

When examining a patient’s spine shape and posture it is important to perform and evaluate quantitative measurements of the curvature of the spine. The spine is physiologically curved in the sagittal plane. However, pathological spinal curvatures in the frontal plane combined with a spinal rotation in the transversal plane are called scoliosis. This is defined as a lateral deviation on the postero-anterior (PA) X-ray image of the spine of more than 10° together along with trunk rotation [1]. The ability to accurately identify the presence of scoliosis in adolescents and interpret the back pain conditions in adults is important in order to provide better treatment, which can consequently improve quality of live.

The most common form of scoliosis is called idiopathic adolescent scoliosis, which affects from 2 to 4 % of the adult population. It is equally common in men and women; however, the probability of an increase in scoliotic curvatures is 10 times higher in women [2]. Scoliosis can cause health problems, such as back pain, and life-threatening functional disorders of the chest organs in cases of severe forms of curvature. Scoliotic curvatures may increase during the growth period; therefore, it is important to monitor the dynamics of the changes. Since 1966, the established method for measuring scoliotic curvature has been a measurement of the Cobb angle on a spinal PA X-ray image of the patient in the upright, standing position [3]. This measurement of the Cobb angle is also used for the sagittal curvature of the spine. Certain X-ray methods can be used to measure the rotation of individual vertebra in the transversal plane [4]. However, repeated X-ray examinations represent a higher risk of cancer genesis [5].

Non-invasive methods, which are harmless to a person’s health, are based on 3D image scanning of the surface of the back and an analysis of the course of the spinous processes. The determined spatial curves of the spine are not identical to the curves determined by the X-ray imaging method; however, they are comparable, which also makes it possible to follow the course of the scoliosis with these 3D methods [6].

A 3D computer image of the surface of the back makes it possible to carry out several analyses. The basis is a reliable determination of the course of the spinous processes, and then further analyses of the acquired spatial spinal curves are possible [7]. In this study we employed the cranio-caudal perspective of the spine, which enabled a horizontal, graphical presentation and a calculation of the deviation of the spine in the sagittal and frontal planes. The aim of this study was to develop a new, non-invasive laser based method for differentiating between scoliotic and healthy subjects which could be useful in clinical work with scoliotic patients.

## Methods

In this research all the participating subjects were distributed in two groups:

- The scoliosis group, consisted of 28 patients (mean age 41.6 years, range 16–82 years, 23 females and 5 males) with clinically confirmed scoliosis
- The control group, consisted of 247 subjects (mean age 42.0 years, range 20–69 years, 123 females and 124 males) with clinically confirmed physiological spinal curvatures

The 3D measurements of the human backs were performed using a 3D laser profilometer, which is based on the translational movement of one laser plane across the measuring surface. The measuring principle of the system is based on laser triangulation [8]. This principle was also used in one of our previous articles [9]. The measuring protocol for each patient was as follows: the patient is measured in the upright, standing position. During each measurement the patient is leaning against some foam that is attached to the wall, with their arms released near the body, while they hold their breath. This posture is one of the four standard positions for the subject that makes it possible to acquire reliable 3D data for the human torso; it is described in the ISO 20685:2010 standard.

In addition, three characteristic anatomical landmarks are manually determined and marked on the back of the individual patient by palpating the spinous process. This task is carried out by a physiatrist:

- C point – the cervicothoracic (C7-Th1) transition spine zone
- Th point – the thoracolumbar (Th12-L1) transition spine zone
- L point – the lumbosacral (L5-S1) transition spine zone

The accuracy of the spinous-processes palpation is estimated from the width of the spinous processes. Based on data found in literature, the accuracy of the palpation was determined to be 9.8 mm in the frontal plane [10].

**Fig. 1:**
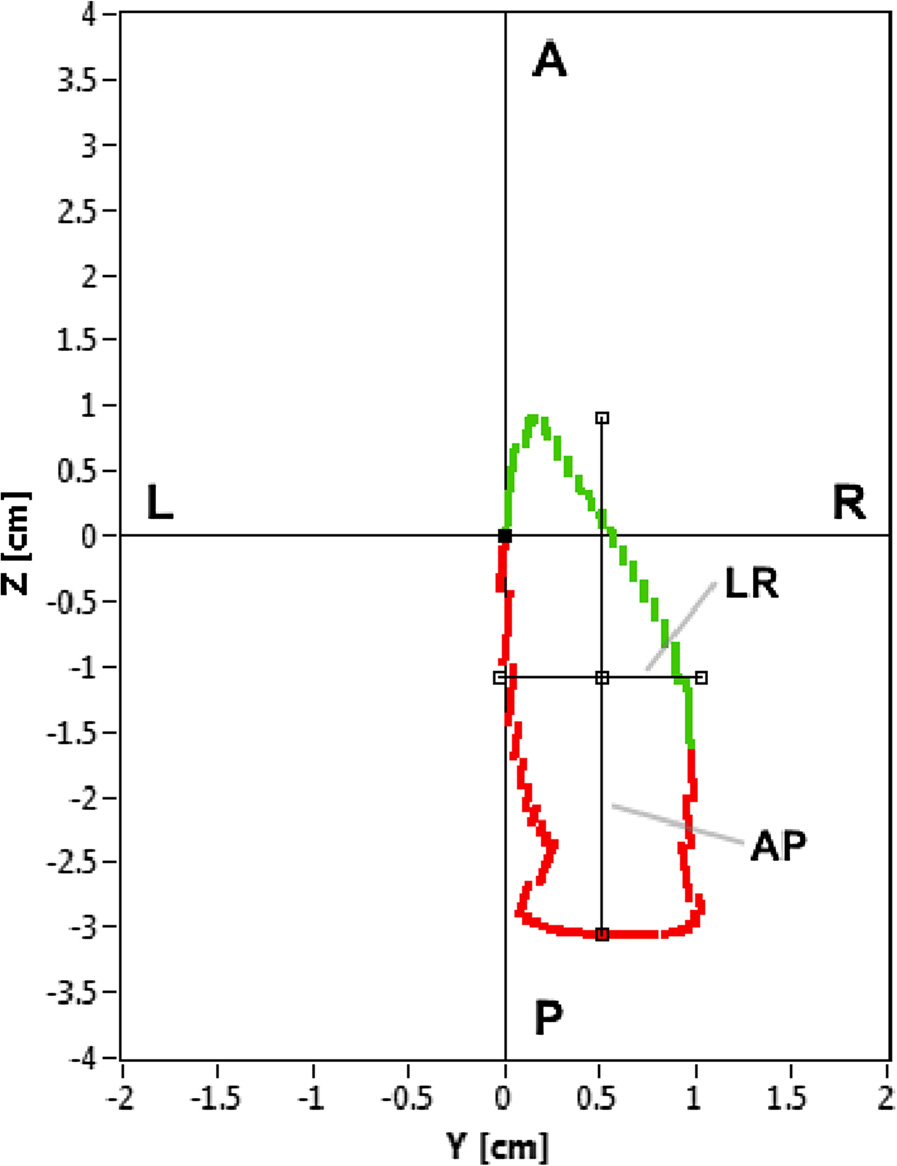
Diagram showing the course of the spine curve in the cranio-caudal view. Besides, the measured distances between the extreme points, denoted as AP and LR are shown. The red-coloured spinal curve denotes the thoracic spinal curve points, while the green-coloured spinal curve denotes the lumbar spinal curve points

The most important procedure step is the determination of the thoracic and lumbar spatial spinal curve between the C and L point by detecting the surface curvature extremes from the 3D measurement of the shape of the back, which is thoroughly described in [7]. In the last step the determined curve of the spine is translated and rotated using the rotation matrix [11]. As a result the curve of the spine is presented in the transversal plane, which is perpendicular to a line segment that is defined by the initial point and the end point of the spinal curve. Curvatures of the spine in the cranio-caudal view are presented by projecting the automatically determined spatial curve of the spine on the diagram shown in Fig. 1 with the following properties:

- The diagram is positioned in a plane that is perpendicular to the CL segment
- The C and L points are positioned at an arbitrary point (0, 0) of the coordinate system
- The abscissa – the axis Y represents the scatter of the spinal curve points in the left-right (LR) view
- The distance between the minimum and maximum points in the left-right view is represented by the LR parameter
- The ordinate – the axis Z represents the scatter of the spinal curve points in the anterior-posterior (AP) view
- The distance between the minimum and maximum points in the anterior-posterior view is represented by the AP parameter
- The course of the spinal curve points is marked by two colours: the thoracic spinal curve points between the C and Th points are marked with red, while the lumbar spinal curve points between the Th and L points are marked with green.

For all the subjects the following parameters were calculated:

- A standardized range of scatter of the spinal curve points in the anterior-posterior view: AP/CL. Since the patients had different body heights, the parameter AP was normalised by dividing by the length of the thoracic and lumbar spine, i.e., the distance between the points C and L.
- A standardized range of scatter of the spinal curve points in the left-right view: LR/CL.
- The value of the range of scatter in the left-right view divided by the range of scatter in the anterior-posterior view LR/AP

All the measured parameters were compared between the scoliotic and control groups using the statistical tests. The test of normality was conducted using the Shapiro-Wilk Test. In order to determine whether two samples of both groups were coming from the same two populations with the same mean, the Student’s *t*-Test was used for the normal data. In the case of the non-normal data, the Kruskal-Wallis test was used as a non-parametric method for testing whether the samples originated from the same distribution.

In order to emphasize the difference between the patients with differently expressed scoliosis, an illustrative comparison with the graphs and the calculated parameters for three subjects are shown. The first patient had physiological spinal curvatures. The second patient had mild scoliosis with a Cobb angle of around 20°, and the third patient had moderately severe scoliosis with a Cobb angle of around 40°. Pictures of the backs with an automatically determined spinous-processes line are presented, together with the diagrams and the calculated parameters.

The presented research was presented to the Republic of Slovenia National Medical Ethics Committee, which stated that there is a compliance with the Helsinki Declaration. The committee made a positive declaration and approved the carrying out of the presented research (reference number 30/02/09). All the patients consented to participate in the presented research and gave an informed written consent also regarding the publication of this paper including accompanying images. In the case of a child’s participation in the research, the informed written consent was obtained from either the parent or the guardian.

## Results

The proposed method, based on laser triangulation principle enables exact determination of the spatial spine curve in the first step. The second step of the method is based on detection and following the spine curvatures. The results of comparison of the AP and LR distances between the maximum points, divided by the CL distance are presented in Table 1. The statistical calculations showed a normal distribution of the results for the AP/CL parameter for both groups (*p* > 0.05). The LR/CL and LR/AP parameters are not normally distributed in both groups (*p* < 0.05).

**Table 1:** The probability of the normally distributed calculated parameters AP/CL, LR/CL and LR/AP in the scoliosis and control groups

	Scoliosis group		Control group	
Parameter	Statistical test	*P* value	Statistical test	*P* value
AP/CL	Shapiro-Wilk	0.95	Shapiro-Wilk	0.42
LR/CL	Shapiro-Wilk	<0.001	Shapiro-Wilk	<0.001
LR/AP	Shapiro-Wilk	<0.001	Shapiro-Wilk	<0.001

The comparison of the range of the calculated parameters of the scatter of the spinal curve points between the observed groups is presented in Table 2. The differences between the average range of the LR/CL parameter in the subjects with scoliosis is statistically significantly larger (*p* < 0.05). The significance of the difference is also confirmed by comparing the average values of LR/AP, which are larger for the patients with scoliosis (*p* < 0.05).

**Table 2:** The average values, the standard deviation and the differences between the calculated parameters (AP/CL, LR/CL and LR/AP) in the scoliosis and control groups

Parameter	Scoliosis group	Control group	Statistical test	*P* value
	Mean ± SD	Mean ± SD		
AP/CL	12.41 ± 4.24	10.99 ± 2.47	Student’s t	0.1
LR/CL	4.33 ± 2.24	1.43 ± 0.55	Kruskal–Wallis	<0.001
LR/AP	0.40 ± 0.28	0.13 ± 0.06	Kruskal–Wallis	<0.001

**Fig. 2:**
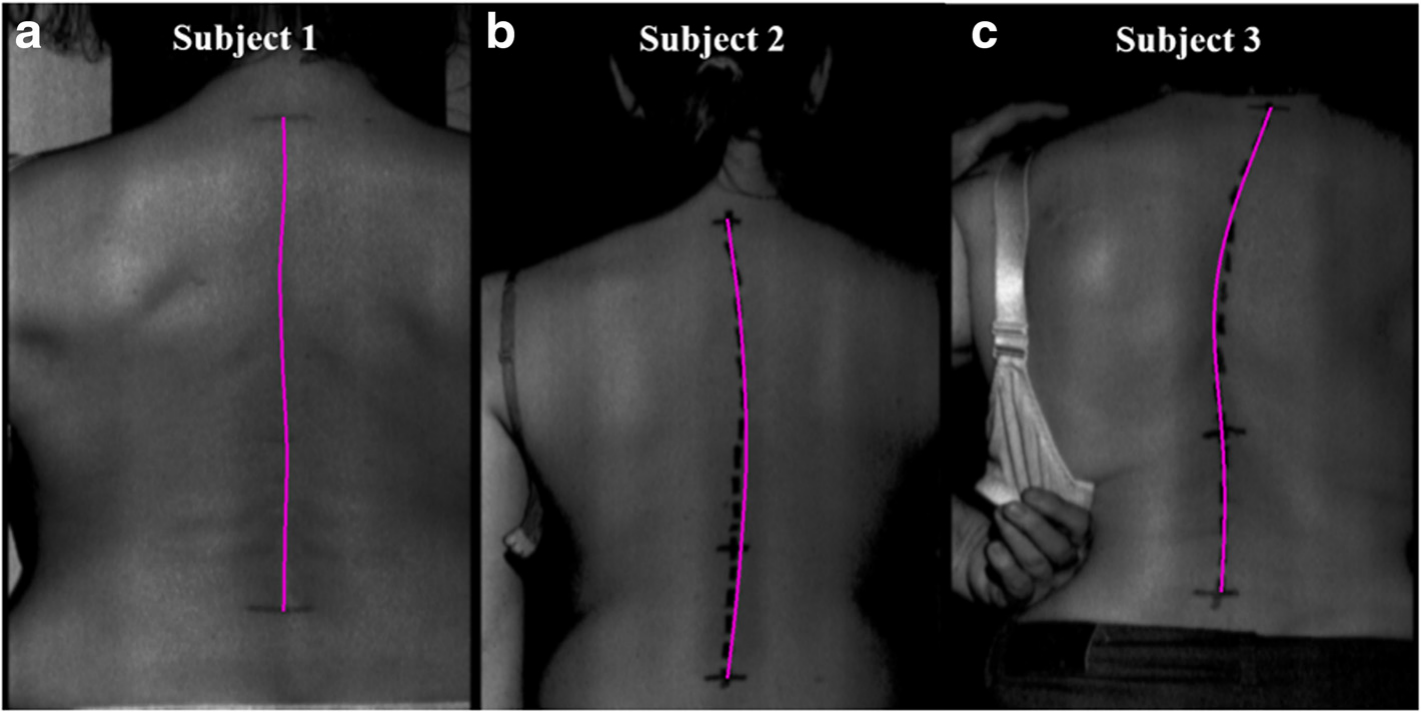
Example of three automatically determined spatial spine curves for the three subjects. Greyscale images of the backs with automatically determined spine curves (purple colour) for the three subjects. **a** Subject 1 – subject with a physiological curve of the spine; (**b**) Subject 2 – subject with mild scoliosis; (**c**) Subject 3 – subject with moderately severe scoliosis

**Fig. 3:**
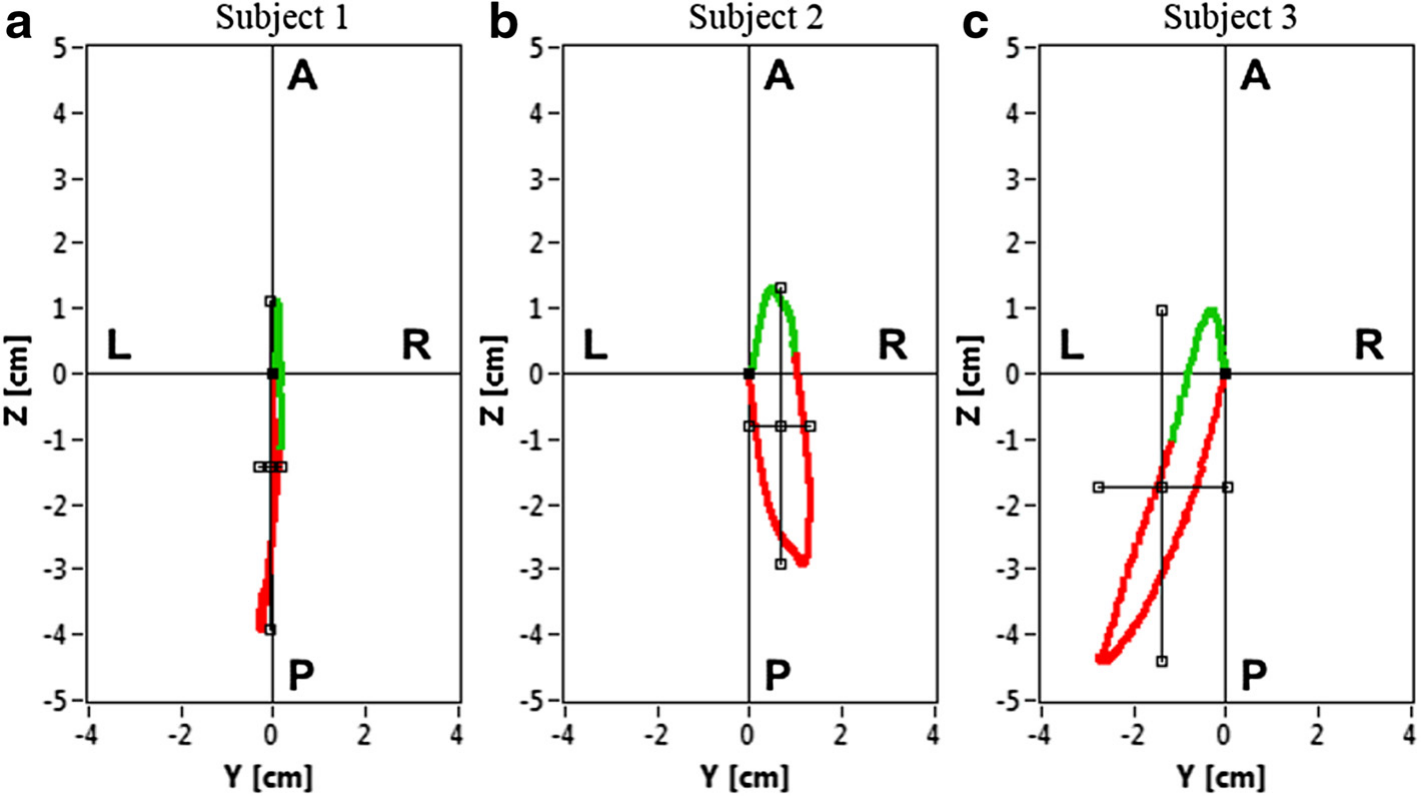
Example of three diagrams of scatter of the spinal curve points for the three subjects. **a** Subject 1 – subject with a physiological curve of the spine; (**b**) Subject 2 – subject with mild scoliosis; (**c**) Subject 3 – subject with moderately severe scoliosis

The applicability of the graphical presentation and the mathematical analysis of the thoracic and lumbar spinal curves are illustrated using the following three examples, shown in Figs. 2, 3 and Table 3.

**Table 3:** Measured and calculated parameters for three subjects

Parameter	Subject 1	Subject 2	Subject 3
Cobb angle [°]	0	20	40
AP/CL [/]	11.98	10.83	12.97
LR/CL [/]	1.01	3.33	7.37
LR/AP [/]	0.08	0.31	0.57
LR [cm]	0.42	1.29	3.05
AP [cm]	5.00	4.21	5.38

Subject 1 – the subject with a physiological spinal curve, Subject 2 – the subject with mild scoliosis, Subject 3 – the subject with moderately severe scoliosis

The LR and AP values are absolute measures that are expressed in centimetres

Based on Figs. 2a, 3a and Table 3 in the case of Subject 1 – the subject with a physiological curvatures of the spine – a large degree of AP scatter can be seen. The scatter in the LR view is minimal, which excludes the important side deviation. The LR/AP quotient shows that the LR scatter is only 8 % of the AP scatter. The red part of the curve indicates the direction and expression of the thoracic kyphosis, i.e., the scatter of the points in the P view, and the green part represents the much smaller lumbar lordosis curve, i.e., the scatter of the points in the A view.

For Subject 2 – the subject with mild scoliosis – the diagram also shows a side scatter of the points in the LR view, which is shown on Figs. 2b, 3b and Table 3. The LR scatter is about 31 % of the scatter in the AP view. The red part of the curve indicates the size of the thoracic kyphosis (P view), which deviates to the right (R view). The green curve presents a relatively smaller AP curve of the lumbar spine, which is in the direction of lordosis (A view), but the deviation is still to the right (R view), i.e., right thoracolumbar scoliosis.

An even more expressed scoliosis is indicated by an even larger increase in the LR scatter. In the case of Subject 3 – the subject with moderately severe scoliosis – the LR scatter is about 57 % of the AP scatter, which is shown on Figs. 2c, 3c and Table 3. The diagram also shows the increased side scatter of points in the LR view. The red part of the curve demonstrates the size of the expressed thoracic kyphosis, which is deviated to the left. The green curve is oriented slightly to the left and the transitions from kyphosis to lordosis are in the direction of the L point.

## Discussion

The paper aims to improve the analysis and quantification of scoliotic curvatures. A scoliotic spine is characterized by a lateral curvature in the frontal plane along with a vertebral rotation in the transversal plane. In terms of different imaging methods and analyses that are frequently used in clinical practise, the deformation is mostly observed and measured based on a posterior and lateral view [6, 12].

The Cobb angle is the established method for measuring the scoliotic curvature and requires an X-ray image of the spine. The Cobb angle defines the deviation in the frontal plane; however, it does not include any information about 3D positional changes of the adjacent vertebrae. In our case the comparison between the method based on Cobb angle determination and the presented method, based on cranio-caudal analysis of the spine curvatures was not performed, since the X-ray imaging method needed for Cobb angle determination was not used in our research. More recent approaches also consider the inclination and deviation of the spine from its natural position in various other positions and provide a cranio-caudal view of the spine [13]; however, it is not quantitatively analysed [12]. The EOS imaging system provides a cranio-caudal view of the spine as a series of vertebrae, which is imposed on the basis, represented as a line between two femoral heads [14]. The vertebrae representing the transitional zones in the scoliosis measurement are marked with a different colour. Since complete vertebras are drawn, the transparency is somewhat reduced and the position of the vertebras is depicted by the pointed vectors. The measurement procedure requires X-ray imaging, with a reduced intensity of ionizing radiation; however, the real risk reduction for the patients has not yet been proven [15].

New, non-invasive, 3D methods, based on optical metrology, allow us to analyse the course of spinous processes, which indirectly show the course of the spine and kyphosis or lordosis of the spine in the sagittal plane and the scoliosis in the frontal plane. The spatial distribution of the points in both planes can be provided by the third dimension – the plane perpendicular to the longitudinal course of the spine. The view in this plane makes it possible to present the whole thoracic and lumbar spine in a simple, clear and informative graphical way. The diagram enables measurements of the most extensive range of points in the AP and LR views. The range of points in the AP view provides the size of the sagittal curves, whereas the LR view provides the scoliotic curvatures. The quotient of both ranges shows the proportion of the LR range in the AP range. The study has shown a statistically significant higher LR/AP quotient (*p* < 0.001) in the subjects with scoliosis (0.40 ± 0.28) compared to the subjects with physiological spinal curvatures (0.13 ± 0.06). A comparison of the acquired results and the data available in the literature was not possible because a review of the medical records showed that such an approach to calculating scoliosis was not used.

To compare the range of scatter of the spinal points in the AP and LR views between the differently sized subjects we standardized the measurement by considering the length of the observed spine, i.e., the length CL. Both points were positioned at the starting point of the diagram to make the diagram more illustrative. In subjects with physiological spinal curvatures, the median drops from C1 onto the S1 plate, the CL line being vertical [16]; therefore, the projection of the spine curve is positioned in the horizontal plane. In the case of scoliosis, the direction of the CL line deviates to some extent because the C point is not projected vertically above the L point. The projection of the spine curve is therefore positioned in the plane, which is not completely horizontal. Consequently, the information of the spatial position of the C and L point is lost, but the display still clearly demonstrates the scatter of the points in the AP and LR views and the presence of the scoliosis.

## Conclusion

The main innovation of the proposed method is the ability to differentiate between scoliotic and healthy subjects, based on cranio-caudal analysis of the spatial spine curve, determined by laser triangulation. The method showed statistically significant differences between the scoliosis and the control group. In addition, longitudinal monitoring of developed scoliotic curvatures of spine in individuals can be performed. This can be beneficial in the process of making decisions for referring a patient to more accurate as well as more aggressive diagnostic procedures, which show the course and 3D orientation of the vertebrae in a scoliotic spine. Therefore, the proposed method can be useful for human posture diagnostics and for monitoring scoliotic spine curvatures in preventive and curative clinical practice at all levels of health care.

## Abbreviations

PA: Postero-anterior
AP: Antero-posterior
LR: Left-right
